# Autophagy and the pancreas: Healthy and disease states

**DOI:** 10.3389/fcell.2024.1460616

**Published:** 2024-09-24

**Authors:** Zixian Zhou, Pengcheng Zhang, Juan Li, Jiaqi Yao, Yuhong Jiang, Meihua Wan, Wenfu Tang, Ling Liu

**Affiliations:** ^1^ Department of Neurology, West China Hospital, Sichuan University, Chengdu, China; ^2^ West China Center of Excellence for Pancreatitis, Institute of Integrated Traditional Chinese and Western Medicine, West China Hospital, Sichuan University, Chengdu, China; ^3^ Regenerative Medicine Research Center, Sichuan University West China Hospital, Chengdu, Sichuan, China

**Keywords:** autophagy, pancreas, zymophagy, organelle homeostasis, genetic studies

## Abstract

Macroautophagy/autophagy is an intracellular degradation pathway that has an important effect on both healthy and diseased pancreases. It protects the structure and function of the pancreas by maintaining organelle homeostasis and removing damaged organelles. A variety of pancreas-related diseases, such as diabetes, pancreatitis, and pancreatic cancer, are closely associated with autophagy. Genetic studies that address autophagy confirm this view. Loss of autophagy homeostasis (lack or overactivation) can lead to a series of adverse reactions, such as oxidative accumulation, increased inflammation, and cell death. There is growing evidence that stimulating or inhibiting autophagy is a potential therapeutic strategy for various pancreatic diseases. In this review, we discuss the multiple roles of autophagy in physiological and pathological conditions of the pancreas, including its role as a protective or pathogenic factor.

## 1 Introduction

Intracellular components are not constant but rather are in a state of dynamic equilibrium. Organelles and proteins are constantly produced, while dysfunctional or redundant components are removed. At least two intracellular cycling pathways are known: the ubiquitin‒proteasome system and the autophagy system (Shrestha et al., 2020). The former degrades ubiquitin-labelled proteins; target proteins are degraded by the proteasome in combination with many enzymes (Zhang et al., 2020). The latter, autophagy, involves the digestion of cytoplasmic components and organelles through lysosomes (Lee A. et al., 2022). In addition, autophagy helps to remove misfolded and aggregated proteins and plays an important role in tissues with high protein synthesis rates, such as the pancreas (Hinzman et al., 2022; Ramachandran et al., 2021; She et al., 2021).

Even without any external stimulation, autophagy, which is called basic autophagy, still occurs in pancreatic cells (Antonucci et al., 2015). Basic autophagy occurs at a low level and is rapidly activated in response to cellular stress, such as hunger (Chediack et al., 2012), oxidation (Pérez et al., 2015), endoplasmic reticulum (ER) stress (Yazıcı et al., 2023; Lugea et al., 2011), or destructive stimulation (Ito et al., 2009; Telbisz et al., 2002). This response is usually beneficial, helping pancreatic cells cope with environmental stress and avoid death. However, in the absence of autophagy, this protective mechanism is not activated. For example, blocking pancreatic autophagy has been shown to increase the sensitivity of mice to bacterial lipopolysaccharide, and more severe vacuolization and inflammation of pancreatic cells have been observed (Xia et al., 2020). This finding suggests that the activation of autophagy limits pancreatic injury. However, autophagy is not always beneficial, and recent studies have shown that excessive autophagy can aggravate pancreatic damage. There is evidence that, compared with that in WT mice, excessive microtubule-associated protein 1 light chain 3 (LC3) in acinar cells in transgenic GFP-LC3 mice destabilizes the autophagy homeostatic state and exacerbates pancreatitis damages (Mareninova et al., 2020).

This article reviews the important role of autophagy in mediating pancreatic homeostasis; discusses the relationship between autophagy and pancreas-related diseases, including pancreatitis, diabetes and pancreatic cancer; and discusses genetic studies that have addressed autophagy.

## 2 Brief introduction to autophagy function and classification

**FIGURE 1:**
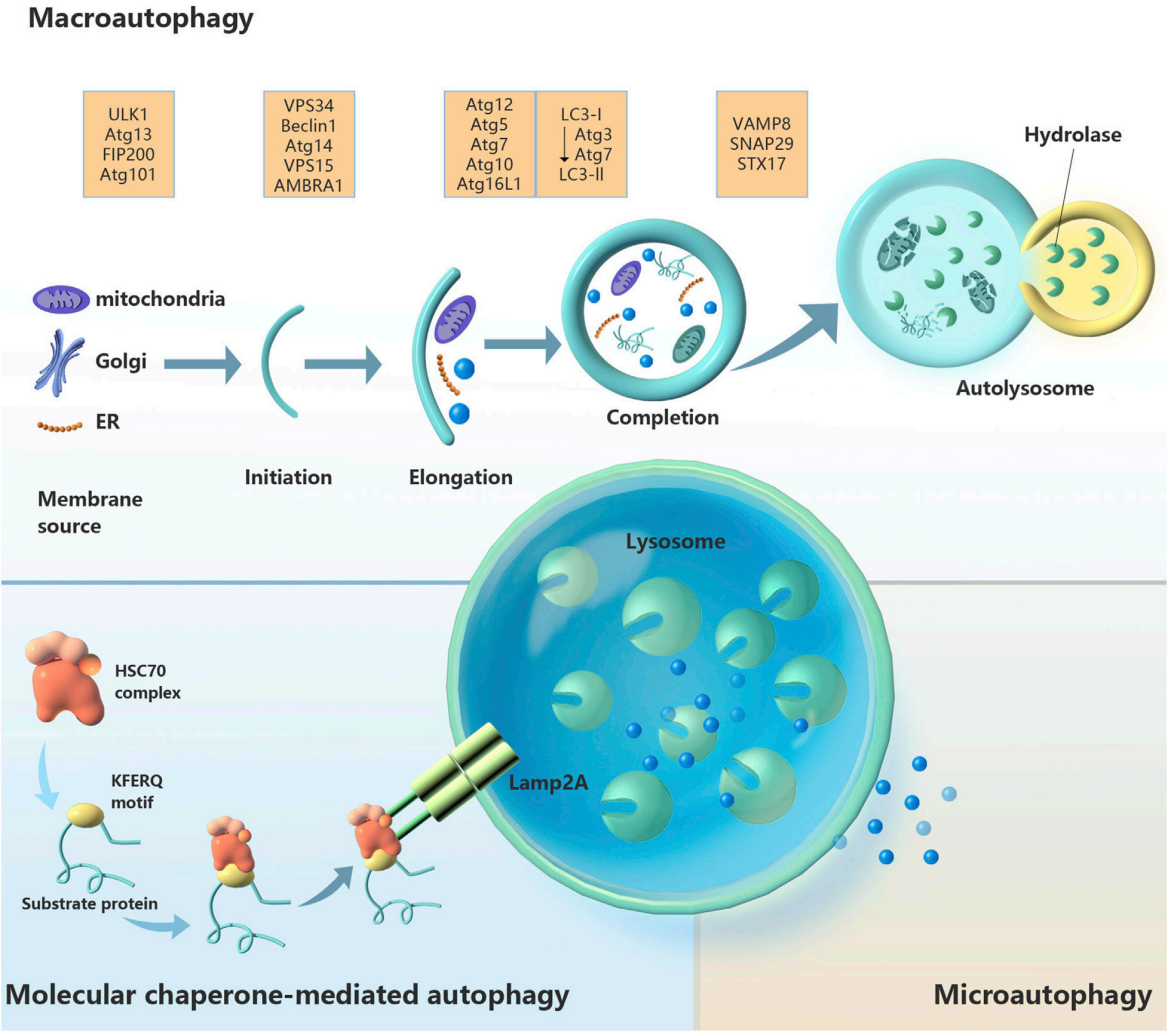
Schematic diagram of the three types of autophagy regulation: macroautophagy, chaperone-mediated autophagy and microautophagy. Abbreviations: AMBRA1, autophagy and beclin 1 regulator 1; Atg, autophagy-related gene; FIP200, focal adhesion kinase family-interacting protein of 200 kD; Hsc70, heat-shock cognate 70; Lamp2A, lysosomal-associated membrane protein 2a; LC3, microtubule-associated protein 1 light chain 3; SNAP29, synaptosome-associated protein 29; Stx17, syntaxin 17; ULK1, unc-51-like autophagy-activating kinase 1; VAMP8, vesicle-associated membrane protein 8; Vps, vacuolar protein sorting.

Autophagy is the biological process by which lysosomes or vacuoles degrade organelles, proteins and other cellular components. The whole process is complex and orderly and is strictly controlled by the synergistic action of at least 30 autophagy-related genes (ATGs) and their products (Zhang et al., 2019). Small-molecule degradation products, such as amino acids and fatty acids, can be recycled (Tang et al., 2022). There are three known types of autophagy: microautophagy, chaperone-mediated autophagy, and macroautophagy (Figure 1). The latter is the most classic type of autophagy. The bilayer membrane structure (also known as the separation membrane or phagocytic mass) appears near the ER. The plasma membrane, ER, Golgi apparatus, recycling endosomes and mitochondria are possible sources of the autophagy membrane (Puri et al., 2014). With the elongation, bending and closure of the membrane structure, autophagosomes are formed. Autophagosomes fuse with lysosomes to form autolysosomes, where the contents are degraded. The second most common type of autophagy is microautophagy, in which lysosomes sag inwards and directly engulf and absorb target cargo (Schuck, 2020). The third type of autophagy, chaperone-mediated autophagy, does not involve membrane recombination; substrate protein containing a specific amino acid sequence (KFERQ) enters the lysosomal membrane via a process mediated by a molecular chaperone (Hsc70) (Diceglie et al., 2021). The most classic and most intensively studied of the above types is macroautophagy, which is referred to as autophagy.

In fact, autophagy has long been considered nonselective. Later studies revealed that autophagy can selectively degrade proteins and organelles (Yang et al., 2019). Damaged organelles rely on autophagy clearance, and different autophagy subtypes can be defined by their degradation products, such as ER-phagy (removal of ER), mitophagy (removal of mitochondria), ribophagy (removal of ribosome), pexophagy (removal of peroxisome), ribophagy (removal of ribosomes) and lipophagy (removal of lipid droplets). Through the above processes, autophagy plays a crucial role in maintaining cell homeostasis and supporting various biological functions in cells.

## 3 Measurement of autophagy activity

The methods used to measure autophagy include static and dynamic methods. The former is still accepted as an index of autophagy activity. Static measurements included Western blot, immunohistochemistry and immunofluorescence analyses of autophagy-related proteins (such as p62 and LC3-II) (Wang Y. et al., 2020; Broggi et al., 2021), transmission electron microscopy (He et al., 2022), assessments of TOR and ATG1 kinase activity (Klionsky et al., 2008), and fluorescence microscopy (Santiago-O’Farrill et al., 2020). LC3-II is generated through lipidation of LC3-I and is subsequently recruited to the autophagosome membrane. Upon fusion of the autophagosome with the lysosome, the autolysosome is formed and LC3-II is delipidated back to LC3-I. Hence, LC3-II is commonly utilized as a marker for autophagy. Additionally, P62 is another autophagy marker, similar to LC3, as it interacts directly with LC3 and undergoes selective degradation within autolysosomes. The expression of autophagy-associated proteins and the state of autophagosomes reflect the transient level of autophagy. A higher LC3-II content, lower p62 content and higher autophagosome content are indicative of higher autophagy levels. However, autophagy is a complex and dynamic process, and sometimes static analysis alone is not enough to truly judge the level of autophagic flux in cells (Liu X. Y. et al., 2022). When the fusion of autophagosomes with lysosomes is inhibited, an accumulation of autophagosomes is noted; nevertheless, autophagic flux is diminished (Lerner et al., 2016). Similarly, rapamycin can increase the expression level of the p62 gene and decrease the level of the p62 protein (Cristofani et al., 2018). In contrast, dynamic measurements tend to analyse autophagic flux, which can more accurately reflect the whole process of autophagy (Chen and Gibson, 2022). For researchers to demonstrate that autophagy is activated, autophagy must be blocked. However, instead, autophagy should be activated to prove that autophagy is blocked. There is no absolute standard for defining the state of autophagy. Therefore, multiangle and dynamic measurements of autophagic flux are helpful for evaluating the autophagy status of cells, tissues or organisms.

## 4 “Zymophagy” - a new selective autophagy pathway

**FIGURE 2:**
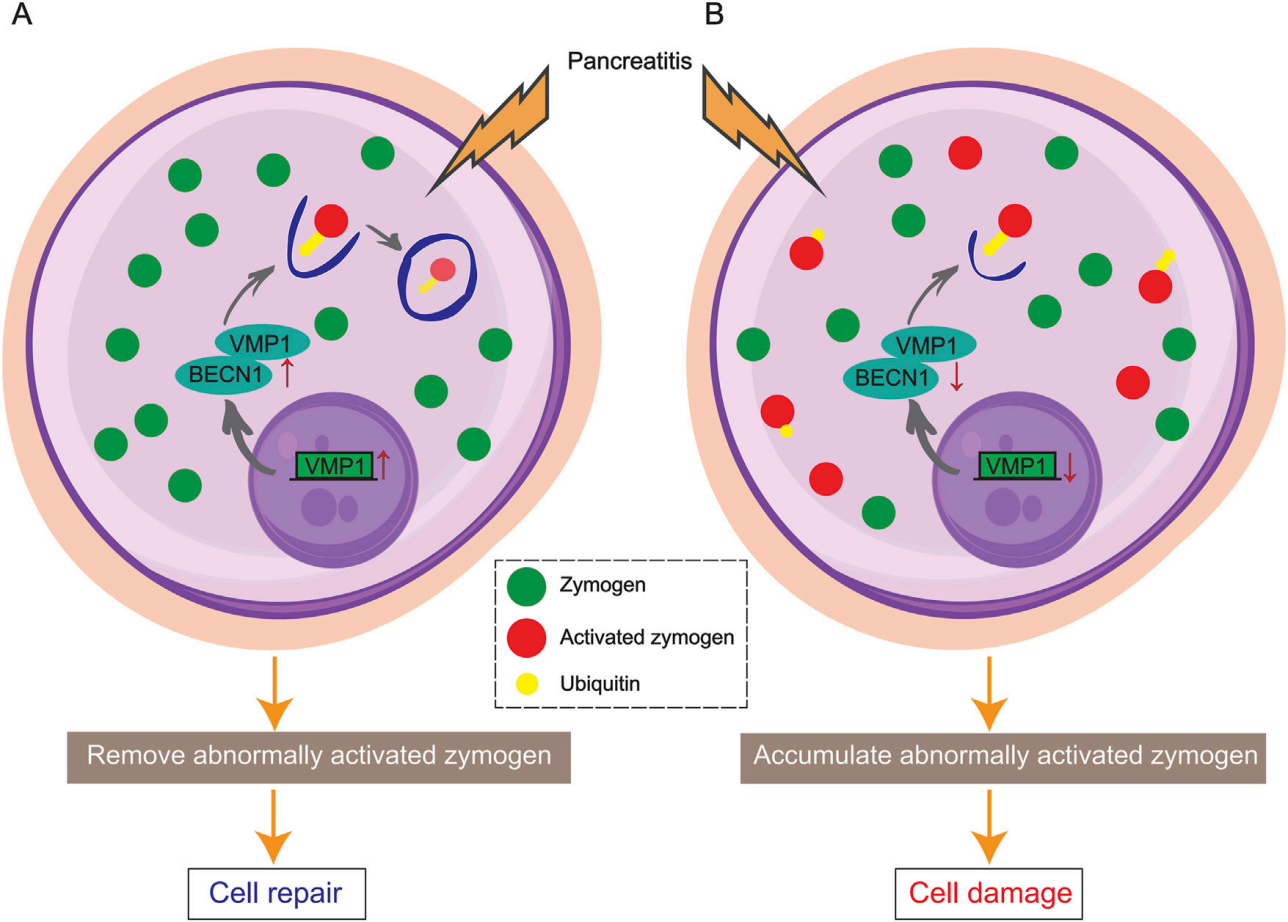
Schematic diagram showing VMP1-mediated zymophagy. **(A)** VMP1 activates and binds to BECN1, triggering zymophagy to remove activated zymogens and repair the pancreas. **(B)** In the absence of VMP1, activated zymogens accumulate in acinar cells, leading to pancreatic damage and pancreatitis.

Zymophagy is a cell rescue mechanism that occurs in acinar cells in response to zymogenic activation (Figure 2) (Wang Q. et al., 2022; Ropolo et al., 2019). Activated forms of zymogens are identified, isolated, and targeted for elimination, thereby decreasing pancreatic injury (Wang et al., 2019; Vaccaro et al., 2022). This protective pathophysiological process may involve complex regulatory mechanisms, but many questions remain unanswered. For example, how does zymophagy occur? How are the activated zymogen granules labeled for zymophagy? Which organelles are involved?

In acinar cells, zymogen ubiquitination is involved in the early zymophagy process. TRIM33 (an E3 ligase) reportedly participates in the ubiquitin modification of trypsin and activates zymophagy (Wang Q. et al., 2022). Following ubiquitination, trypsin binds to vacuole membrane protein 1 (VMP1), a key player in the initiation of autophagy, and is subsequently sequestered to prevent enzymatic damage. VMP1 functions in conjunction with BECN1/Beclin1 to orchestrate this cellular process (Vaccaro et al., 2008; Grasso et al., 2009; Wang S. et al., 2022).

VMP1 was first identified in rats with experimental acute pancreatitis (AP) and has since been the subject of extensive research as a crucial player in zymophagy (Dusetti et al., 2002). In a healthy state, the expression of VMP1 triggers autophagy, but not zymophagy and not pancreatitis. Studies have shown that knocking down VMP1 inhibits the formation of autophagosomes triggered by rapamycin and starvation (Vaccaro et al., 2008). Deletion of VMP1 in mouse acinar cells led to tissue inflammation within 8 days, while knockout of *Atg5* and *Atg7* took longer to induce inflammation (Wang S. et al., 2022). In an acute pancreatitis model induced by stimulation with the cholecystokinin receptor, a change in the localization of VMP1 was observed, as VMP1 translocated from the base of acinar cells to a region rich in zymogen granules (Grasso et al., 2011). Compared with wild-type mice, ElaI-*Vmp1* mice (pancreatic acinar-specific transgenic mice) exhibited significantly reduced cellular inflammation and necrosis after pancreatitis was induced with caerulein (Grasso et al., 2011). In contrast, in the absence of VMP1, acinar cells fail to respond appropriately to spontaneously and prematurely activated zymogen in the pancreas, potentially leading to acinar cell damage and pancreatitis (Wang S. et al., 2022). The reports highlight the positive impact of the VMP1-mediated autophagy pathway and zymophagy, shedding light on the pancreas' self-protection mechanisms in both normal and diseased states.

## 5 Autophagy and pancreatic homeostasis

Here, we explore pancreatic autophagy in the context of energy deficiency and the direct relationship between autophagy and organelle homeostasis to analyse the role of autophagy in different physiological and pathological conditions in the pancreas.

### 5.1 Energy deficiency and autophagy

Autophagy was originally discovered during starvation and is used by cells as a survival strategy when energy is scarce (Wang F. et al., 2020). In a long-term fasting study in birds, autophagy was induced in a variety of digestive organs, and the pancreatic mass decreased by more than 20% (Chediack et al., 2012). In another study, fasting increased the expression of the pancreatic 9cRA protein, which in turn supported the role of FoxO1 in pancreatic β cells, including reducing oxidative stress, promoting autophagy and reducing DNA damage, partly by inducing *Atg7* mRNA (Yoo et al., 2023). Hunger during pregnancy affects not only the pregnant woman but also the foetus. Studies have shown that the consumption of a low-protein diet by mothers enhances neonatal pancreatic autophagy and induces ER stress in β cells (Yang et al., 2020). Although these results fill some gaps, the following questions remain unanswered: What role does β-cell ER stress play in this process? How much crosstalk occurs between ER stress and autophagy, and how does the level of pancreatic autophagy change when the foetus returns to a normal diet after birth? Future research may help in fully understanding the mechanisms underlying the nutritional limitations of autophagy in foetuses.

Generally, hypoxia has been considered one of the means of inducing autophagy (Liu et al., 2019). However, a study using islet cells showed that this is not the case. Hypoxia increases ROS and downregulates the expression of autophagy-related proteins in pancreatic β-cells, and the antioxidant NAC reverses this trend (Wu et al., 2022). Autophagy in pancreatic β cells may play a protective role in hypoxia. Similarly, in pancreatic stellate cells, autophagic flux does not increase due to hypoxia, possibly because cells meet their energy needs in other ways; autophagy is not necessary (Estaras et al., 2022). In fact, it is worth exploring whether more severe oxygen deprivation activates autophagy in pancreatic stellate cells.

### 5.2 ER homeostasis and autophagy

Acinar cells have a very high biosynthesis rate; many proteins are produced in the ER and Golgi apparatus. ER strictly controls the quality of proteins to ensure their correct folding and modification. Under pathological conditions of impaired ER function, the excessive accumulation of unfolded or aggregated proteins leads to ER stress (Zhu et al., 2019). ER stress triggers a series of signalling pathways that relieve ER stress; for example, the unfolded protein response (UPR) is activated, which downregulates general translation and upregulates the transcription of genes that mediate ER stress (Huiting et al., 2018). ER stress and the UPR activate ER-phagy continuously. One study revealed that cell cycle progression gene 1 (CCPG1) is a receptor that mediates ER autophagy (Smith and Wilkinson, 2018a). It binds to ATG8 family proteins and RB1CC1/FIP200 independently and separately, which promotes ER autophagy. CCPG1 drives ER degradation, prevents the excessive accumulation of ER lumen proteins in pancreatic acinar cells, alleviates further UPR production, and ultimately protects the pancreas (Smith and Wilkinson, 2018b; Smith et al., 2018). Accordingly, when these steady-state pathways cannot resolve ER stress, acinar cells tend to undergo apoptosis (Jia et al., 2019).

### 5.3 Mitochondrial homeostasis and autophagy

Healthy mitochondria are essential for the synthesis, storage and secretion of trypsin in pancreatic cells. In mammals, there are two types of mitochondrial autophagy: PTEN-induced kinase 1 (PINK1)-dependent and -independent autophagy (Zhao et al., 2020; Xu et al., 2021). PINK1 is a serine/threonine kinase expressed in mitochondria that plays an important role in initiating mitochondrial autophagy (Li et al., 2019). PINK1-independent autophagy can directly induce mitochondrial degradation, which is mediated by BNIP3/BNIP3L/FUNC1 (Wang Y. et al., 2022).

PINK1 and the E3 ubiquitin ligase Parkin are involved in mitochondrial autophagy. Under basic conditions, PINK1 is transferred to the mitochondrial inner membrane, where it is then rapidly cleaved and degraded by the protease presenilin-related rhomboid-like (PARL) (Kim et al., 2022; Sonn et al., 2022). PINK1 in dysfunctional mitochondria accumulates in the outer membrane and is activated by autophosphorylation at Ser228 to recruit and activate Parkin (Rasool et al., 2018). After Parkin activation, many protein substrates are ubiquitinated, and autophagy receptors (such as OPTN and NDP52) are recruited (Li et al., 2022). Two ubiquitin positive feedback circuits regulate PINK1/Parkin-mediated mitochondrial autophagy: phosphorylated ubiquitin or the ubiquitin-like Atg8 protein family (Padman et al., 2019). Then, autophagy signal activation initiates mitochondrial autophagy and clears damaged mitochondria.

Although PINK1 has been widely studied as a major regulator of mitochondrial autophagy, recent studies have shown that PINK1 does not play an important role in basic mitochondrial autophagy. In *Pink1*-KO mice, only pancreatic islet tissue exhibited changes in basal mitophagy due to the loss of *Pink1*; other tissues were not affected (McWilliams et al., 2018). The possible reason is that the regulation of mitochondrial autophagy is complex and environment dependent and that there is a PINK1-independent pathway involved in basic mitochondrial autophagy. There is a compensatory increase in PINK1-independent mitochondrial autophagy in response to PINK1 deletion. Notably, the increased level of basic mitochondrial autophagy in islets may be due to the activation of compensatory autophagy signals and help to relieve metabolic pressure (McWilliams et al., 2018). However, the following questions remain: What is the precise function of PINK1 in mammalian mitochondria? How does the compensatory mechanism of PINK1-independent mitochondrial autophagy work?

### 5.4 Lysosomal homeostasis and autophagy

The central role of lysosomes in autophagy has long been known. Autophagy depends on the effective fusion of lysosomes and autophagosomes to remove damaged or ageing proteins (Gao et al., 2020). Once the lysosome itself is damaged, the damage can be sensed by galactose lectin, which is subsequently recruited to the lysosome to repair or remove the damaged lysosomal membrane (Hu et al., 2022). Galactose lectins include galactose lectin-3 (Gal-3) and galactose lectin-9 (Gal-9). At the molecular level, Gal-9 and lysosome-associated membrane protein 2 (LAMP-2) have been shown to play important roles in maintaining lysosomal homeostasis and pancreatic autophagy, as well as in preventing pancreatic disease (Sudhakar et al., 2020). Gal-3 has been shown to be associated with pancreatic cancer autophagy. For example, Gal-3 deficiency leads to a decrease in LC3 levels in pancreatic cancer cells (da Silva Filho et al., 2020). LAMP-2 is a major membrane protein component and is involved in the occurrence and maintenance of lysosomes (Mareninova et al., 2015). Pathological changes in lysosomal membrane proteins can lead to lysosomal dysfunction. In addition, LAMP-2 is a key protein that mediates autophagy-related lysosome formation, and its depletion impairs autophagic flux (Li et al., 2018).

## 6 Autophagy and pancreatic physiology

The importance of autophagy in maintaining pancreatic homeostasis in the physiological environment has been elucidated in experimental animals via genetic changes in autophagy (Table 1). These experimental models involving deletion of autophagy-related genes may provide insights into the mechanistic role of autophagy in pancreatic health and disease.

**TABLE 1:** Genetic studies investigating the relationship between autophagy and pancreatic physiology *in vivo*.

Model	Intervention	autophagy flux	Observations	References
Mice	*Atg5* ^−/−^	↓	Zymogen activation decreased and mitigated pancreatitis injury induced by caerulein	Ohmuraya and Yamamura (2008)
Mice	*Atg5* ^−/−^	↓	Destruction of pancreatic endocrine cells	Yang et al. (2014)
Mice	*Atg5* ^−/−^	↓	Pancreatic cell necrosis; inflammation; acinus-ductal metaplasia and hypertrophy; and pancreatic atrophy and degeneration	Diakopoulos et al. (2015)
Mice	*Atg7* ^−/−^	↓	Islet degeneration and impaired glucose tolerance; Increased number of acinar cell zymogen particles; and persistent cell death	(Ebato et al., 2008), (Iwahashi et al., 2018)
Mice	*Atg7* ^−/−^	↓	Induced acinar cell dedifferentiation to form ductal progenitor cells and promote acinar regeneration	Antonucci et al. (2015)
Mice	*Atg7* ^−/−^	↓	Significantly increased sensitivity to endotoxin-induced pancreatitis	Xia et al. (2020)
Mice	*Atg7* ^−/−^ *Rip3* ^−/−^	↓	Loss of *Rip3* further accelerated the internal and external secretion dysfunction caused by *Atg7* loss	Zhou et al. (2017a)
Mice	*Becn1^−/−^*	↓	The mouse pancreas was almost completely lost	Mehanna et al. (2023)
Mice	*Ctsb* ^−/−^ *Ctsd* ^−/−^	↓	CP phenotype and impaired autophagy of pancreatic acinar cells	Sendler et al. (2018)
Mice	*Errγ* ^−/−^	↓	Pancreatic acinar cells were deficient in energy, with disrupted autophagy and REDOX homeostasis	Choi et al. (2022)
Mice	*Fam134b* ^−/−^	↓	Compared with WT mice, knockout mice were more susceptible to starvation-induced pancreatic damage	Keles et al. (2020)
Mice	*GFP-LC3* ^TG^	↑	Exacerbated autophagy damage in experimental pancreatitis	Mareninova et al. (2020)
Mice	*Gnptab* ^−/−^	↓	Pancreatic inflammation and the dysregulation of cholesterol metabolism	Mareninova et al. (2021)
Mice	*Hmgb1* ^−/−^	↓	Pancreatic inflammation; elevated serum amylase; acinar cell death; and interstitial oedema	Kang et al. (2014)
Mice	*Igf1r* ^+/−^	↑	The ageing of pancreatic β-cell was inhibited, accompanied by improved glucose tolerance	Iwasaki et al. (2023)
Mice	Irf2^−/−^	↑	The exocytosis of pancreatic acinar cells was impaired, and the pancreas became resistant to pancreatitis induction	Mashima et al. (2011), Mashima et al. (2020)
Mice	*Lamp-2* ^−/−^	↓	Accumulation of pancreatic autophagy vacuoles	Eskelinen et al. (2002)
Mice	*Lat1* ^−/−^	↓	*Lat1* knockout slowed the recovery of exocrine function after AP induction, an effect that was sex-dependent	Hagen et al. (2022)
Mice	*Lmna* ^−/−^	↑	ER stress with pancreatic exocrine dysfunction and CP-related phenotypes	Elenbaas et al. (2018)
Mice	miR-155 coding adenovirus	↓	Inhibited pancreatic autophagy in AP and reduced inflammation	Wan et al. (2019)
Mice	*Munc18c* ^+/−^	↑	Reduced caerulein-induced pancreatic injury	Dolai et al. (2018)
Mice	*Pkd3* ^−/−^	↑	Ameliorated caerulein- and L-arginine-induced pancreatic injury	Yuan et al. (2021)
Mice	*Pink1* ^−/−^	↑	Basal mitophagy changed in pancreatic islets	McWilliams et al. (2018)
Mice	*Prkci* ^−/−^	↓	Promoted immune cell infiltration and cell apoptosis in the pancreas	Inman et al. (2022)
Mice	*Rab7* ^−/−^	↓	Delayed endosome and autophagosome maturation and impaired lysosome function	Takahashi et al. (2017)
Mice	*Rab9* ^TG^	↓	Pancreatic inflammation; acinar cell necrosis; and apoptosis	Mareninova et al. (2022)
Rat	*Snap23* encoding adenovirus	↓	Disrupted autophagosome and lysosome fusion but not autophagosome development; helped prevent pancreatitis	Dolai et al. (2021)
Mice	*Spink3* ^−/−^ *Spink3* ^+/−^	↑	Complete loss of *Spink3* resulted in acinar cell death and impaired pancreatic integrity, and no significant acinar cell regeneration was observed; a small presence was enough to prevent pancreatitis	Ohmuraya et al. (2012), Ohmuraya et al. (2005), Romac et al. (2010)
Mice	*Stx17* encoding adenovirus	↓	Aggravated symptoms of caerulein-induced pancreatitis	Wang et al. (2023)
Mice	*Tfeb* ^−/−^ *Tfe3* ^−/−^	↓	Spontaneous occurrence of pancreatitis	Wang et al. (2019)
Mice	*Ubiad1* ^−/−^	↑	Pancreatic atrophy and acinar cell disappearance	Nakagawa et al. (2019)
Mice	*Vamp8* ^−/−^	↓	Resistant to caerulein- or alcohol-induced pancreatitis	Messenger et al. (2017)
Mice	*Vmp1* ^−/−^	↓	Pancreatic inflammation; acinar cell death; and fibrosis	Wang et al. (2022b)
Mice	*Vmp1* ^TG^	↑	Induced autophagosome formation in pancreatic cells	Ropolo et al. (2007)

AP, acute pancreatitis; *Atg5*, autophagy related 5; *Atg7*, autophagy related 7; *Becn1*, Beclin1; CP, chronic pancreatitis; *Ctsb*, cathepsin B; *ctsd*, cathepsin D; *Errγ*, oestrogen-related receptor γ; *Hmgb1*, high mobility group box 1; *Igf1r*, insulin like growth factor receptor; *Irf2*, interferon regulatory factor-2; *Lamp-2*, lysosome-associated membrane protein 2; *Lmna*, Lamin A/C; *Pkd3*, protein kinase D 3; *Pink1*, PTEN-induced kinase 1; *Prkci*, protein kinase C iota; *Rip3*, receptor interacting protein 3; *Snap23,* synaptosome-associated protein 23; *Spink3*, serine protease inhibitor Kazal type 3; *Stx17*, Syntaxin17; *Tfeb*, transcription factor EB; *Ubiad1*, UbiA prenyltransferase domain-containing protein 1; *Vamp1*, vesicle-associated membrane protein 1; *Vamp8*, vesicle-associated membrane protein 8.

The premature activation of trypsinogen by lysosomal hydrolase is a characteristic event and key step in the occurrence of pancreatitis, and one of the important influencing factors is the inhibition of digestive enzyme secretion. In acinar cells, the secretion of zymogen granules is regulated by vesicle-associated membrane proteins (VAMPs), including VAMP2 and VAMP8 (which mediate early secretion and mid-late secretion, respectively). A sharp decrease in VAMP8 leads to the accumulation of intracellular trypsin and the loss of endosomes, which are potential mechanisms of pancreatitis (Pilliod et al., 2022). *Vamp8* ^−/−^ mice were protected from CCK-8-induced zymogen accumulation and acinar damage, suggesting that CCK-8-induced pancreatitis inhibits VAMP8-related zymogen secretion rather than VAMP2-related zymogen secretion (Messenger et al., 2017). It is reasonable to believe that maintaining VAMP8-dependent pro-enzyme secretion helps to reduce the accumulation and activation of proenzymes during pancreatitis, which is one of the potential strategies for reducing pancreatitis injury. Furthermore, the intravenous injection of a miR-155-encoded adenovirus has been shown to alleviate pancreatic injury by reducing the accumulation of autophagosomes in AP mouse cells (Wan et al., 2019). Similarly, in *Atg5*-deficient mice, zymogen activation was reduced in the pancreatic acini due to a lack of autophagy, thereby alleviating caerulein-induced pancreatitis injury (Ohmuraya and Yamamura, 2008). Furthermore, another study has shown that pancreatic damage induced by caerulein or alcohol is limited in mice intravenously injected with encoding adenovirus for synaptosome-associated protein of 23 (SNAP23) (Dolai et al., 2021).

A trypsin inhibitor, serine protease inhibitor Kazal type 3 (SPINK3) is essential for the integrity of the pancreas, and its absence leads to excessive autophagy in acinar cells (Ohmuraya et al., 2012). *Spink3* ^−/−^ mice die of pancreatic autophagy within a few days after birth, surviving less than 15 days (Ohmuraya et al., 2005). Although SPINK3 plays an important role in maintaining acinar autophagy and cell homeostasis, a low level of this protein seems to be sufficient to prevent pancreatitis. A small amount of SPINK3 expression in *Spink3* ^+/−^ mice did not affect the increased sensitivity to experimental pancreatitis (Romac et al., 2010). The pancreas of adult mice undergo significant changes when the UbiA prenyltransferase domain-containing protein 1 (*Ubiad1*) is systemically knocked out. These changes include pancreatic atrophy and loss of acinar cells, and are accompanied by enhanced autophagy (Nakagawa et al., 2019). Laminin is associated with pancreatitis because mice without lamin A/C (LMNA) in the pancreas exhibit ER stress, pancreatic exocrine dysfunction and a series of CP-related phenotypes (Elenbaas et al., 2018).

Although the above results suggest that autophagy contributes to the occurrence of pancreatic injury, a large amount of evidence shows that autophagy plays an indispensable role in maintaining pancreatic homeostasis. For example, endocrine cell destruction and a range of similar tissue manifestations, such as inflammation, necrosis, acinar-ductal metaplasia and hypertrophy, and pancreatic atrophy and degeneration, were observed in mice with a deletion of pancreas-specific Atg5 (Yang et al., 2014; Diakopoulos et al., 2015). Consistent with these findings, *Atg7*-deficient mice also exhibited impaired endocrine systems and reduced insulin secretion (Ebato et al., 2008). In addition, protective and anti-inflammatory effects of autophagy in the pancreas have been confirmed. An increase in the number of acinar cell zymogen granules and persistent cell death were observed in Atg7-deficient mice with pancreatic autophagy deficiency, and these mice exhibited significantly increased sensitivity to endotoxin-induced pancreatitis (Iwahashi et al., 2018; Chen et al., 2022). It has been reported that the deletion of receptor interacting protein 3 (*Rip3*, a necroptotic signalling factor) exacerbates the acinar loss caused by *Atg7* deficiency and is related to immune cell infiltration (Zhou et al., 2017a). The pancreas-specific knockdown of syntaxin17 (Stx17) exacerbates the symptoms of caerulein-induced pancreatitis, which is associated with the disruption of protective autophagy (Wang et al., 2023). In addition, Farnesoid X receptor (FXR) plays a protective role in pancreatitis by restoring pancreatic autophagy through the enhancement of Oxidative stress-induced growth inhibitor 1 (OSGIN1, a tumor suppressor) (Zheng et al., 2022). Pancreatic loss of *Fxr* increases the sensitivity of mice to acute and chronic pancreatitis induced by caerulein, but GW4064 (an agonist of FXR) limits pancreatic damage (Zheng et al., 2022). The Overexpression of interleukin-22 (IL-22, an inflammation-related factor) significantly alleviates pancreatic necrosis, apoptosis and tissue inflammation induced by caerulein (Feng et al., 2012). Notably, *Atg7* deletion also triggers a regeneration mechanism that induces acinar cells to dedifferentiate into ductal progenitor cells, which contributes to the recovery of acinar tissue function (Antonucci et al., 2015). However, LAT1 promotes the regeneration of pancreatic cells after AP in a sex-dependent manner (Hagen et al., 2022).

Many zymogen granules were observed in the cytoplasm of *Irf2* ^−/−^ mouse acinar cells, indicating that IRF2 is a key factor in mediating zymogen-related exocytosis, at least in acinar cells (Mashima et al., 2011). In addition, *Irf2* ^−/−^ acinar cells can partially resist the induction of pancreatitis, an effect that is related to the significant upregulation of the calcium-binding proteins S100 g and Anxa10 (Mashima et al., 2020). Another study also revealed that *Munc18c* ^+/−^ mice lacked basolateral exocytosis of zymogen granules and exhibited mild pancreatitis under caerulein overstimulation (Dolai et al., 2018). The above results suggest that zymogen granule exocytosis may play a more important role in the occurrence of pancreatitis than the increase in the number of autolysosomes. Further exploration of the exocytosis of these zymogen granules will help in obtaining a more comprehensive understanding of pancreatitis.

The absence of protein kinase D3 (*Pkd3*) in the pancreas promotes autophagy and limits injury in experimental pancreatitis (Yuan et al., 2021). The inhibition of typical (LC3-mediated) autophagy and activation of atypical (RAB9-mediated) autophagy were observed in mice overexpressing RAB9, which resulted in pancreatitis-like damage (Mareninova et al., 2022). RAB9-mediated atypical autophagy cannot completely replace LC3-mediated classical autophagy, at least in pancreatic cells. In fact, VMP1 also affects the homeostasis of pancreatic acinar cells. VMP1 was conditionally overexpressed in mouse pancreatic acinar cells, which induced the production of many vacuoles in acinar cells (Ropolo et al., 2007). However, mice with *Vmp1* deletion in pancreatic acinar cells rapidly develop pathological changes similar to those observed in human chronic pancreatitis (Wang S. et al., 2022).

Caerulein-induced autophagy deficiency in acinar cells is due to a decrease in the number of lysosomes, which may be related to the fact that caerulein induces mTOR and promotes the degradation of the transcription factor EB (TFEB) (Wang et al., 2019). A decrease in nuclear TFEB in acinar cells was observed in a mouse model of pancreatitis, an effect consistent with what has been observed in human pancreatitis. *Tfeb* ^−/−^ mic*e* exhibit spontaneous severe pancreatitis and pancreatic fibrosis, and worsen caerulein-induced experimental pancreatitis (Wang et al., 2019). In contrast, mice overexpressing TFEB demonstrate a protective effect against alcohol-induced pancreatic tissue damage (Wang S. et al., 2020). Because lysosomal biogenesis depends on TFEB, activating TFEB to enhance lysosomal activity is likely a possible strategy for the prevention and treatment of pancreatitis.

The role of LAMP-2 in autophagy has been studied in *Lamp-2*-deficient mice (Eskelinen et al., 2002). LAMP-2 deficiency directly or indirectly leads to the accumulation of autophagic vacuoles in multiple tissues, including the pancreas. Similarly, mice lacking protein kinase C iota (PRKCI, a serine/threonine protein kinase) in the pancreas exhibit autophagic destruction of acinar cells, which promotes pancreatic immune cell infiltration and apoptosis (Inman et al., 2022). Similarly, pancreatic-specific *Rab7* deletion hinders the progression of autophagy to autophagic lysosomes and affects endosome maturation and endocytosis, which leads to more severe tissue inflammation (Takahashi et al., 2017). The relationship between cathepsin and autophagy has also been reported recently. The results showed that neither cathepsin B (CTSB) nor cathepsin D (CTSD) alone could cause autophagy damage (Sendler et al., 2018). However, mice with simultaneous deletions of *Ctsb* and *Ctsd* exhibited impaired autophagy, indicating that both co-regulate pancreatic autophagy (Sendler et al., 2018). Similarly, trypsin activity increases during AP in *Ctsb-* and cathepsin L (*Ctsl*)-knockout mice (Chen et al., 2022).

Bmp4 is a protein that regulates insulin synthesis, processing, and transport. The results from transgenic mice showed that autophagy is also involved in the response of the pancreas to hunger (Yasunaga et al., 2011). Interestingly, starvation for 36 h induces pancreatic damage in *Fam134b* ^−/−^ mice but not in WT mice (Keles et al., 2020). Senescence is related to the functional degradation of tissues and cells. A recent study reported that the deletion of *Igf1r* in the pancreas of mice inhibits pancreatic β-cell senescence, accompanied by improved glucose tolerance (Iwasaki et al., 2023). In addition, *Gnptab* ^−/−^ mice spontaneously develop pancreatitis and cholesterol metabolism disorders (Mareninova et al., 2021). Consistent with these findings, energy deficiency in pancreatic acinar cells and loss of autophagy and redox homeostasis were observed in oestrogen-related receptor γ (*Errγ*) conditional knockout mice (Choi et al., 2022).

## 7 Autophagy and pancreatic pathology

In numerous animal models of pancreatic disease, the modulation of autophagy, whether through pharmacological or genetic interventions, is linked to alterations in disease severity or progression (Table 2).

**TABLE 2:** Pharmacological and genetic studies examining the relationship between autophagy and pancreatic pathology *in vivo*.

Model	Intervention	Observations	References
AP/CP (Mice)	*Fxr* ^*−/−*^	Knockout mice were more sensitive to acute and chronic pancreatitis induced by caerulein and pancreatic tissue oedema and necrosis	Zheng et al. (2022)
AP (Mice)	*IL-22* ^*TG*^	Significantly improved caerulein-induced AP and reduced pancreatic necrosis, apoptosis and tissue inflammation	Feng et al. (2012)
Pancreatitis (Mice)	*Tfeb* ^*−/−*^	Spontaneous occurrence of severe pancreatitis and pancreatic fibrosis	Wang et al. (2020c)
Pancreatitis (Mice)	*Tfeb* ^*−/−*^ *Tfe3* ^*−/−*^	Aggravated pancreatic injury	Wang et al. (2019)
Diabetes (Rat)	Dioscin	Reduced pancreatic damage was correlated with autophagy regulation	Zhong et al. (2022)
Diabetes (Mice)	Rehmanniae Radix	Improved function was correlated with increased levels of autophagy	Yan et al. (2023)
Diabetes (Mice)	*Fat-1* ^*TG*^	Enhanced beta cell basal autophagy and alleviated streptozotocin-induced diabetes	Hwang et al. (2015)
Diabetes (Mice)	*hIAPP* ^*TG*^	β-cell ER stress and impaired autophagy occurred during pregnancy	Gurlo et al. (2019)
Diabetes (Mice)	SGY-P	Reduction in pancreatic β-cell apoptosis was correlated with the regulation of autophagy	Xing et al. (2020)
Diabetes (Mice)	C3G	The pancreatic protective effect of C3G treatment was related to mitophagy	Ye et al. (2021)
PC (Mice)	*Kras* ^*G12D*^	Persistent pancreatic inflammation and an increased incidence of cancer	Chang et al. (2017)
PC (Mice)	*Pdx1-Cre Kras* ^*G12D/+*^ *Hmgb1* ^*−/−*^	Accelerated the progression of pancreatic ductal adenocarcinoma	Song et al. (2018)

AP, acute pancreatitis; CP, chronic pancreatitis; *Fxr*, farnesoid X receptor; *hIAPP*, human islet amyloid polypeptide; *Hmgb1*, high mobility group box 1; IL-22, interleukin-22; Pdx1, pancreatic and duodenal homeobox gene 1; *Tfeb*, transcription factor EB; SGY-P, sangguayin preparation; C3G, cyanidin-3-O-glucoside; PC, pancreatic cancer.

### 7.1 Diabetes

The pancreas is a key organ for regulating carbohydrate metabolism, especially glucose metabolism. The pancreas secretes two hormones that are essential for the regulation of blood sugar: insulin and glucagon. Glucagon is the main hormone that increases blood sugar, while insulin is the only hormone with a hypoglycaemic effect in animals (Gong et al., 2019). Islet β-cells are the only source of insulin in the blood, and their dysfunction or loss can lead to diabetes (Le et al., 2022). Studies have shown that diabetes can in turn induce pancreatic tissue cell senescence (Xue et al., 2022). The pathogenesis of diabetes is complex, causing damage to the pancreas itself, and is accompanied by changes in multiple systems throughout the body, including nutritional and metabolic disorders, tissue ageing, hormone level changes (Ruze et al., 2023).

Islet amyloid polypeptide (IAPP) is secreted by islet β cells, and its misfolding or aggregation is related to β cell loss and stress. Human *IAPP* (*hIAPP*) transgenic mice exhibited pregnancy-induced ER stress and autophagy damage (Gurlo et al., 2019). This study revealed the pathogenesis of gestational diabetes mellitus associated with autophagy. Db/db mice, characterized by a lack of leptin receptors, spontaneously develop type 2 diabetes and oxidative stress damage in the islets (Xing et al., 2020). Cyanidin-3-O-glucoside (C3G, a plant extract) restores pancreatic function in diabetic mice by activating partial autophagy of mitochondria (Ye et al., 2021). Compared with those of control mice, mice overexpressing Fat-1 exhibited greater levels of basic autophagy in β cells and reduced diabetic damage induced by streptozotocin (Hwang et al., 2015).

The role and mechanism of islet β-cell apoptosis in diabetes have been widely studied. However, the role and mechanism of islet β-cell autophagy in diabetes are still unclear. (Grasso et al., 2009) used streptozotocin (STZ), which is a drug that selectively targets and destroys islet β cells, to establish an experimental diabetes model. According to their results, autophagy is triggered in the early cellular events of diabetes in STZ-induced rats, VMP1 activation occurs in islet cells and the interaction of VMP1-BECN1 stimulates this process (Grasso et al., 2009). Notably, STZ may stimulate islet cells to produce ROS through autophagy, because ROS regulate the activity of ATG4, a key factor in autophagy (Grasso et al., 2009; Lee A. R. et al., 2022). These results suggest that autophagy may contribute to STZ-induced β-cell damage; however, the role of autophagy in diabetes appears to be contradictory. Autophagy may also play a protective role during diabetes, limiting rather than exacerbating cell death. Autophagy plays an active role by removing damaged organelles and ageing cells. For example, autophagy helps clear diabetes-induced damaged mitochondria (Wang et al., 2017). When autophagy is disrupted, cell homeostasis and stress responses become uncontrolled (Muralidharan et al., 2021).

Controlling autophagy is a very promising strategy for the treatment of diabetes. Increasing the level of autophagy can effectively reduce oxidative stress and apoptosis in experimental diabetic animals (Zhu X. et al., 2021; Wang et al., 2018; Liu C. et al., 2022). Oxidative stress is considered the key factor in diabetic injury, and autophagy protects cells from oxidative stress by degrading oxidative stress products. Therefore, drugs like dioscin (Zhong et al., 2022) and Rehmanniae Radix (Yan et al., 2023) can strongly reduce diabetic damage by activating autophagy. In addition, the activation of autophagy can relieve pressure on the ER and improve mitochondrial function (Zhong et al., 2022). Moreover, the protection of cells from apoptosis reduces the inflammatory response and dysfunction.

### 7.2 Pancreatitis

#### 7.2.1 Increase in autophagosomes but inhibition of autophagy flux

Since Chiari proposed the scientific hypothesis of pancreatitis, it has been generally accepted that pancreatitis is caused by the abnormal activation of zymogens within the pancreas (Dolai et al., 2021; Huang et al., 2020). The pathogenesis of pancreatitis is complex. Early cellular events include dysfunctional autophagy, the pathological exocytosis of zymogen granules, and the activation of trypsin and IKKβ (Saluja et al., 2019). A recent study reported a common component involved in these three events, namely, soluble N-ethylmaleimide-sensitive factor attachment receptor (SNARE) proteins, which include Munc18c and STX17 (Dolai et al., 2021). These proteins are located in the plasma membrane under physiological conditions and are then transferred to autophagosomes during pathological conditions, where they mediate pathological basolateral exocytosis and IKKβ-mediated autolysis (Dolai et al., 2018; Dolai et al., 2021).

Experimental pancreatitis models constructed with cholecystokinin, caerulein, alcohol and Coxsackie virus have been used to simulate human pancreatitis and explore the aetiology of the disease (Han et al., 2022; Kim et al., 2019). In these models, vacuoles accumulate in acinar cells, mainly autophagosomes or autolysosomes, usually with large volumes (Mareninova et al., 2021). The accumulation of large vacuoles in acinar cells observed via histology or transmission electron microscopy provides strong evidence for the diagnosis of pancreatitis. The number and size of autophagic vacuoles and the levels of the autophagy-associated proteins p62 and LC3-II are increased in WT mice (Mareninova et al., 2020). These results reflect the impaired autophagic flux in pancreatitis, findings that are consistent with what has been observed in human disease (Biczo et al., 2018). Several studies have further revealed the relationship between autophagy and pancreatitis; for example, in GFP-LC3 transgenic mice with induced pancreatitis, more intense autophagosome accumulation was observed, which was associated with more severe tissue damage (higher serum amylase levels) (Biczo et al., 2018). Researchers have also shown that bone morphogenetic protein in the pancreas is associated with the production of vacuoles in acinar cells and elevated LC3-II levels (Cao et al., 2013).

#### 7.2.2 Regulating autophagy: a good method for treating pancreatitis

Based on the above analysis, autophagy dysregulation is a key event in pancreatitis. Therefore, one reasonable strategy is to restore blocked or disrupted autophagy to an efficient and unobstructed state. The formation and degradation of autolysosomes are critical for autophagosome clearance, and lysosomal abnormalities have been found in both experimental pancreatitis models and human pancreatitis patients (Mareninova et al., 2021; Şentürk et al., 2019; Chen et al., 2020). Therefore, increasing the number and function of lysosomes, promoting the fusion of autophagosome and lysosome, and enhancing the degradation of autolysosome may contribute to alleviating pancreatic injury. The rationality of this view is supported by several studies (Wang et al., 2023; Zheng et al., 2022; Li et al., 2020). Notably, moderate levels of autophagy, rather than blindly activating autophagy, are beneficial. In fact, the inhibition of overactivated autophagy also helps to combat pancreatitis (Feng et al., 2012). Overall, although the mechanism of autophagy in pancreatitis is unclear, regulating autophagy is one of the most promising strategies for the treatment of this disease.

### 7.3 Pancreatic cancer

Pancreatic cancer is a highly lethal tumour characterized by strong proliferation, high invasion and multiple drug resistance (Boukrout et al., 2021). Autophagy plays a dual role in pancreatic cancer and is highly involved in its occurrence and development (Wei et al., 2019). On the one hand, autophagy, as a protective mechanism, is used to maintain cell homeostasis and genomic stability and prevent normal cells from transforming into malignant cells (Rahman et al., 2022). Without autophagy, toxic and harmful components (such as damaged organelles and damaged proteins) cannot be removed from pancreatic cells, leading to oxidative stress and subsequent DNA damage (Zhou et al., 2017b). On the other hand, autophagy is involved in the occurrence of pancreatic cancer and serves as a survival strategy for tumour cells to help them respond to environmental stress (Iovanna, 2017; Molejon et al., 2018).

The occurrence of pancreatic cancer is closely related to the presence of the *Kras* oncogene. *Kras* mutations are associated with the epithelial–mesenchymal transition (EMT) process and mediate cell carcinogenesis and cancer metastasis. An increasing number of studies have shown that autophagy plays a pivotal role in the malignant transformation of pancreatic cells mediated by *Kras* mutation. It has been reported that the overexpression of VMP1 exacerbates the tumour-promoting effect of *Kras*, an effect that can be reversed by chloroquine (an autophagy inhibitor) (Iovanna, 2017). Increased expression of the PRKCI in *Kras* ^G12D^ transgenic mice (often used as an experimental animal model of pancreatic carcinogenesis), a finding that is consistent with what has been observed in human disease (Scotti et al., 2012). The deletion of pancreatic PRKCI blocks the autophagy of acinar cells and the transition from pancreatic intraepithelial neoplasia to pancreatic cancer (Inman et al., 2022). Notably, despite the presence of carcinogenic *Kras* expression, the transformation of pancreatic cells to eventual pancreatic cancer is limited (Todoric et al., 2017). These findings show that additional regulatory pathways are involved in the development of pancreatic cancer. This complex process includes a series of specific factors, such as p53 mutation (Yang et al., 2014), p62 accumulation (Todoric et al., 2017), lncRNA regulation (Shafabakhsh et al., 2021), the antioxidant stress response (Gong et al., 2016) and the activation of inflammatory signalling pathways (Iovanna, 2016).

KCH (*Pdx1*-Cre;*Kras* ^G12D/+^;*Hmgb1* ^−/−^) mice were developed as a model of accelerated pancreatic ductal adenocarcinoma progression (Song et al., 2018). Oral JTC801 (a compound with strong anticancer activity) effectively limits tumour growth and changes the expression of autophagy-related markers (Song et al., 2018). High-fat, high-calorie diet (HFCD) feeding significantly increased the incidence of cancer in *Kras* ^G12D^-expressing mice, an effect that was associated with persistent pancreatic inflammation and autophagy disorders (Chang et al., 2017).

In both animal and human specimens, cancer tissue has greater autophagy activity than does surrounding tissue (Hirayama et al., 2009). When pancreatic cell lines are exposed to hypoxia, autophagy helps to increase cell survival (Owada et al., 2017). Similarly, pancreatic cancer also depends on autophagy for survival in the absence of nutrients (Yuan et al., 2022; Zhou et al., 2022). In other words, pancreatic cancer cells can use autophagy to cope with nutritional limitations caused by strong cell proliferation. In addition, autophagy is also a means by which pancreatic cancer cells escape immunosuppression (Zhu X. G. et al., 2021) and the action of drugs (Liu et al., 2014). However, further studies are needed to clarify whether these tumours promote autophagy-related processes via unique or shared potential mechanisms.

It is thought that the role of autophagy in different stages of tumorigenesis is dynamic. In pancreatic cells, autophagy disorders can cause genomic disorders and PanIN lesions (Yang et al., 2014). However, more precancerous lesions do not transform into more pancreatic cancer cells but rather into fewer cells. Blocking autophagy in pancreatic cancer not only suppresses the survival of cancer cells but also helps to disrupt autophagy in pancreatic cancer stem cells, thus enhancing the therapeutic efficacy of anticancer drugs (Leng et al., 2021). Therefore, autophagy is an important target for the treatment and prevention of pancreatic cancer. However, little is known about the specific mechanisms by which autophagy acts as both a tumour suppressor and a tumour enhancer in pancreatic cancer, and elucidating these mechanisms will be helpful for improving treatment strategies for individuals with pancreatic cancer.

## 8 Conclusion and future prospects

**FIGURE 3:**
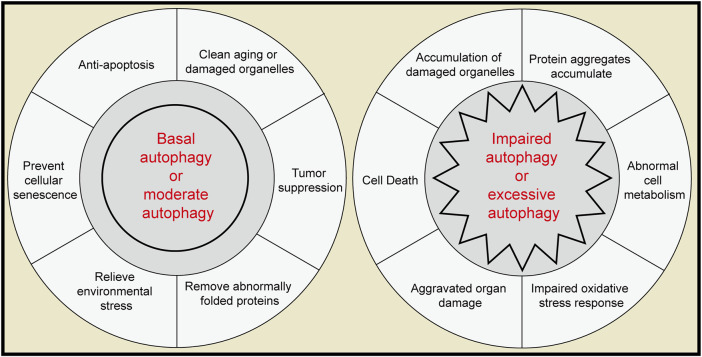
The role of autophagy in the pancreas. Left, moderate autophagy acts as a protective factor in the pancreas. Right, extreme autophagy can act as a pathogenic agent in the pancreas.

Autophagy plays an important role in the physiology and pathology of the pancreas and plays different roles in different stages (Figure 3). Basic autophagy and moderate autophagy in response to environmental stress are considered protective and protect pancreatic cells from dysfunction and apoptosis. Extreme levels of autophagy (overactivation or autophagy disorder) can become destructive and may mediate more severe cell death and tissue damage. Numerous questions remain unresolved, particularly regarding the impact of other forms of autophagy, such as microautophagy and chaperone-mediated autophagy, on pancreatic function. Equally important, additional types of selective autophagy need to be considered, including fat autophagy, ribosomal autophagy and peroxidase autophagy. For example, ferritin autophagy has been shown to help promote the survival of pancreatic cancer and help it acquire therapeutic resistance (Jain and Amaravadi, 2022).

In fact, autophagy in the pancreas is pluripotent, and its mechanism may be closely and complexly related to the life processes of other cells. Future research will help in better understanding pancreatic autophagy and adjusting treatment strategies.

## Figures and Tables

**Table:** 

**AMBRA1**	autophagy and beclin 1 regulator 1
**AP**	acute pancreatitis
**ATG**	autophagy-related gene
**Becn1**	Beclin1
**CP**	chronic pancreatitis
**CTSB**	cathepsin B
**Ctsd**	cathepsin D
**ER**	endoplasmic reticulum
**ERRγ**	oestrogen-related receptor γ
**FIP200**	focal adhesion kinase family-interacting protein of 200 kD
**FXR**	farnesoid X receptor
**Gal-3**	galactose lectin-3
**hIAPP**	human islet amyloid polypeptide
**HMGB1**	high mobility group box 1
**HSC70**	heat-shock cognate 70
**IGF1R**	insulin like growth factor receptor
**IL-22**	interleukin-22
**IRF2**	interferon regulatory factor-2
**LAMP-2**	lysosome-associated membrane protein 2
**LC3**	microtubule-associated protein 1 light chain 3
**LMNA**	Lamin A/C
**PKD3**	protein kinase D 3
**PINK1**	PTEN-induced kinase 1
**Prkci**	protein kinase C iota
**RIP3**	receptor interacting protein 3
**SNAP23**	synaptosome-associated protein 23
**SPINK3**	serine protease inhibitor Kazal type 3
**STX17**	Syntaxin17
**TFEB**	transcription factor EB
**UBIAD1**	UbiA prenyltransferase domain-containing protein 1
**ULK1**	unc-51-like autophagy activating kinase 1
**VAMP1**	vesicle-associated membrane protein 1
**VMP1**	vacuole membrane protein 1
